# The utilization of artificial intelligence applications to improve breast cancer detection and prognosis

**DOI:** 10.15537/smj.2023.44.2.20220611

**Published:** 2023-02

**Authors:** Walaa M. Alsharif

**Affiliations:** *From the Diagnostic Radiology Technology Department, College of Applied Medical Sciences, Taibah University, Al Madinah Al Munawwarah; and from the Society of Artificial Intelligence in Healthcare, Riyadh, Kingdom of Saudi Arabia.*

**Keywords:** artificial intelligence, breast cancer, breast imaging

## Abstract

Breast imaging faces challenges with the current increase in medical imaging requests and lesions that breast screening programs can miss. Solutions to improve these challenges are being sought with the recent advancement and adoption of artificial intelligent (AI)-based applications to enhance workflow efficiency as well as patient-healthcare outcomes. rtificial intelligent tools have been proposed and used to analyze different modes of breast imaging, in most of the published studies, mainly for the detection and classification of breast lesions, breast lesion segmentation, breast density evaluation, and breast cancer risk assessment. This article reviews the background of the Conventional Computer-aided Detection system and AI, AI-based applications in breast medical imaging for the identification, segmentation, and categorization of lesions, breast density and cancer risk evaluation. In addition, the challenges, and limitations of AI-based applications in breast imaging are also discussed.

**B**reast cancer is the second biggest cause of death for women, and early screening and identification can increase treatment choices and reduce mortality rates.^1-3^ Consequently, several countries have applied breast screening programs for all women between the ages of 40 and 50. Over 42 million exams are undertaken across the world.^4-6^ It was reported that breast screening programs can miss between 15% and 35% of cancers, either due to error or because the cancer was not detectable or perceptible to radiologists at the time of scanning.^7^ Also, radiologists may face challenges in breast image interpretation, as breast images are subjected to several limitations. First, breast density can affect image sensitivity, which may result in breast cancer not being detected during the screening.^8,9^ Second, false-positive findings can be generated, which leads to patient discontent and increased cost and workload. These false-positive findings can lead to unnecessary follow-ups and invasive diagnostic practices such as biopsies.^10,11^

Computer-aided detection (CAD) has been designed and used in breast imaging to aid radiologists and automate the early discovery and diagnosis of breast lesions.^12^ Studies show that a solo interpretation plus CAD can be used as an substitute to double reading.^13,14^ However, several studies highlight the low specificity of the CAD systems and no improvement in cost-effectiveness.^15,16^ Increasing call-back rates/false-positive recalls were found when CAD had been used in breast screening.^17^ This inaccuracy and unpredictability of the CAD system has raised some doubts about whether artificial intelligence (AI) applications and recent advances in deep learning (DL) can help radiologists improve their performance in detecting breast cancer.^18^ Machine learning (ML) and DL are both subsets of AI. The ML model requires more human interference and structured data to achieve results. It should be provided with data/inputs, image features (scuh as shape, width, and edge), applying a classification algorithm, and then the model will predict outputs. However, the DL model permits direct features extraction from the original unstructured data/inputs (such as image, text). Therefore, the DL model outweighs the ML model in the case of large datasets as no manual feature extraction is needed.^19^ A DL-CAD tool has been recently developed and used for breast lesion detection and characterization (benign or malignant).^20-25^ The literature refers to the potential role of DL-CAD regarding enhancing diagnostic accuracy and specificity in breast screening. Despite the AI strengths reported in the literature, several drawbacks of the existing AI applications have also been highlighted (Table 1).^26^ This review aims to assess the available literature to evaluate the readiness of the existing AI-based applications for breast screening and to advise the research directions in this field.

**Table 1:** - Strengths and drawbacks of artificial intelligence (AI)-based applications in radiology.

Strengths	Drawbacks
Enhance analysis: Automated pathology screening, detection, and characterization.	Time and cost consuming in training and testing AI model.
Accurate classification: Categorize image based on abnormality (benign and malignant).	Ethical and legal issues: Ethics of data (How should we use, label and protect data?) Ethics of algorithm and trained model (How does the AI model make decisions? How can we diminish the risk of patient harm from privacy breaches? Who is responsible for mistakes resulting from the use of the AI model?) Ethics of practice (monitor and verify AI-driven autonomy)
Extract additional required information from previous detected pathology.	Biased predicted outcomes due to incomplete and/or unrepresentative data.
Offer a second opinion which increases confidence of the diagnosis.	Lack of interpretability can lead to a lack of trust and acceptance of AI models by physicians and patients.
Minimize interindividual variability, bias and time.	*Lack* of strong evidence and regulations to support the *use of an AI model.*
	Lack of standardized benchmarks which make it difficult to validate the performance of an AI model.

## Conventional Computer-Aided Detection system (CAD) and AI-based applications

The role of computers in assisting radiologists in their clinical practice is not new. In 1998, CAD systems utilizing traditional ML were developed and used as a second opinion to analyze patients’ images in mammography and improve radiologists’ performance.^27,28^ The effectiveness of this instrument in breast cancer diagnosis has long been controversial. Literature shows that despite the positive impact of CAD systems on breast cancer screening, decreases in specificity and increases in recall rates are also noted. Several studies failed to ascertain the meaningful value of the CAD system in clinical practice. The relationship between CAD system usage, image interpretation accuracy, and recall rates has been documented in the literature.^15^ Lehman et al^17^ claim that there is no improvement in detection rate and/or prognostic characterization of breast cancer with the CAD system.

The conventional CAD system has been established to aid radiologists, not to be used as a primary screening tool. It is designed and trained to detect specific features that radiologists look for, such as masses or classifications. This conventional system depends on manual features extraction by expert.^29^ Although the conventional CAD system can achieve a high sensitivity compared to radiologists, high false-positive rates might increase.^30^ Therefore, radiologists should screen and read the images as carefully as they would without CAD, and then use CAD as ‘spell checker’ following their own interpretation.^28^ This tool has evolved over time from traditional approaches to modern DL methods. Deep learning-based CAD can interpret vast amounts of data, learn features from images, learn from mistakes, and improve performance over time.^31^ This makes DL technology more robust to adapt different circumstances related to the type of scanners and patient population (when the training data are available).^28^ Currently, conventional/artificial neural networks (CNNs/ANNs) are most commonly used in DL for pattern recognition tasks in images.

**Figure 1:**
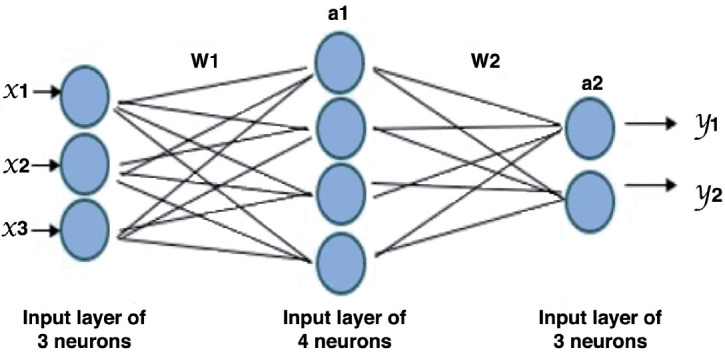
- Conventional/Artificial neural networks (CNN/ANN) composition

The human brain is made up of millions of neurons, so CNNs are really just a composition of perceptions, connected in different ways and operating on different activation functions. Conventionals basically process information in a similar way to the human brain: they have self-learning capabilities, and will learn from examples (such as inputs/images) and experience (such as training) without the need for manually designed features.^32^ They are composed of fully connected layer, which creates the features map and passes it next to a pooling layer, where down-sampling takes place, then the outcome is passed to a fully connected layer to be assorted; and finally the output layer, which creates the analysis results of the data (Figure 1).^33^ The addition of layers is determined by the application and the problem that needs to be solved. The more layers there are, the more feature extraction and data abstraction can be achieved.^34^ The potential role of deep CNNs (DCNN) brings strong guarantees in improving the accuracy and promoting CAD as a clinical support system for medical imaging.^28^

## AI-based applications in breast cancer screening

The DCNNs can be designed and applied to detect lesions (such as changes in size, shape, or texture), classifications (such as benign/malignant), and segmentation of organs and tumors.^18,35^ This section will discuss the current advancement of DL for breast screening and cancer detection in different imaging modalities.

## AI-based application and mammography

Mammography is a non-invasive method that is often used to detect breast cancer.^36^ It can successfully identify non-palpable masses and classify between benign lesions and malignant tumors.^37^ The CAD system was developed to facilitate decision-making and to lessen the demand for numerous readers. Using the CAD system, on the other hand, has been linked to higher recall rates in breast cancer screening.^14,16^ Notable advancements have been made in recent years on AI-based applications to analyze mammographic images to detect breast masses and calcifications.^38-40^ A higher performance was shown by radiologists in cancer detection as measured by the area under the receiver operating characteristic curve (AUC) when using AI in reduced reading times (reduced by approximately 4.5%).^39^ A comparable result was stated by Pacile et al.^40^ This might imply that AI will take over the more routine and tedious cases, so that radiologists have sufficient time to focus on complex cases.^39^ A dramatic improvement in the detection of breast cancer was noted among radiologists with less experience and trainees when they used the AI.^39,41^ A reasonable explanation for this finding may have to do with a greater acceptance of AI-based applications by younger radiologists. In addition to this, experienced radiologists may ignore the flags produced by the AI more often than those with less experience.^41^ Dembrower et al^42^ studied the potential change in cancer detection and found that AI application can detect subtle tumors that were not identified previously without AI. Another study has recently been published in which AI was shown to be superior to radiologists in detecting cancer in cases of fatty and dense breast tissue.^43^ Dahlblom et al^44^ carried out a study to compare the performance of an AI tool with digital breast tomosynthesis (DBT) in the breast cancer detection. They demonstrated that 44% of the breast cancer detected only using DBT by radiologists were spotted with digital mammography by the AI tool.^44^ Although almost half of the cases were identified with digital mammography by AI, further investigation with a larger sample may be warranted.

Researchers refer to the potential advantage of AI tools in finding tumors with marginal indications or false-negative interval cancers on earlier screening exams that have not yet been studied.^45-47^ They discovered that AI tools could help experts in identifying up to 19.3% of the interval cancers with minimal signs of malignancy.^45^ Numerous research compared AI performance to radiologist accuracy in clinical settings. Schaffter et al^48^ stated that AI-based application combined with a radiologist showed higher accuracy in breast image screening in comparison with a single radiologist interpretation alone. This is in line with Salim et al,^49^ who used three commercial AI systems and indicated that one AI application had higher sensitivity (81.9%) and the other two lesser sensitivity (67%, 67.4%) compared to the radiologist (77.4%).^49^ Likewise McKinney et al’s^49^ study found that the AI tool beat the radiologist performance in sensitivity (56% versus [vs] 48%) and specificity (84% vs 81%).^46^ This was in line with Hmida et al^50^ and Sapate.^51^ Even though the aforementioned studies highlight the potential benefit of AI in improving breast cancer screening and image interpretation, it is also important to note that mammographic images were examined in laboratory settings with a limited number of readers, which makes it difficult to generalize the findings to the clinical practice.

## AI-based application and ultrasound (US)

Ultrasound frequently serves as an additional scanning modality to mammography in breast screening programs. It is a common simple imaging technique involved in evaluation of palpable breast abnormalities and characterized breast masses.^52^ Ultrasound scanning of the breast has several benefits related to other imaging modalities, including lower cost, lack of ionizing radiation, and the capability to assess images in real time.^53^ However, radiologists face challenges in interpreting breast US images due to blurry borders, inherent low contrast, and high levels of shadowing.^54,55^ Several researchers referred to intra-reader variability in interpreting breast US images, and increased false-positive findings.^56,57^

Recent advances in DL have accelerated the development of AI-based applications for the automated identification, segmentation, extraction, and classification of breast cancer from US images.^58-60^ A study conducted by Shen et al^61^ to identify malignant lesions in breast US images found that AI tools reach a higher area under the receiver operating characteristic curve (AUROC) and area under the precision-recall curve (AUPRC) than expert radiologists. In the same study, radiologists managed to reduce false positive rates by 37.3% and the number of biopsies requested by 27.8%, while maintaining the level of sensitivity.^61^ Several studies proved that DL won in terms of classification and recognition of breast cancer based on US images.^62-64^ A study carried out by Fujioka et al^59^ highlighted the fact that DL with CNNs showed equal or higher AUC than radiologists in distinguishing benign from malignant breast lesions on US images.^65^ Furthermore, it was found that DL systems showed high performance in breast lesion calcifications with an accuracy of 93.4%, a sensitivity of 88.6%, a specificity of 97.1%, and an area under the AUC of 0.947.62 Similar results were reported by Becker et al^62^ and Han et al,^64^ in which AI systems achieved higher performance in differentiating breast lesions in less time with accuracy similar to that of radiologists.Another AI application was assessed by Mango et al,^66^ who highlighted an improvement in the accuracy of US breast lesion assessment when combining radiologists’ evaluation with the AI. There was a significant drop in the inter- and intra-observer variability.

An AI system equipped in US machines has also been recently proposed, which can offer immediate decision of benignity or malignancy in the static US images after marking the region of interest (ROI).^18^ Kim et al^24^ evaluated the diagnostic functioning of an AI system equipped in a US machine to discern between benign and malignant breast lesions. Accuracy was significantly higher, and the AUC was 0.72 compared to the radiologists. A year later in 2018, a similar performance was indicated by Di Segni et al,^67^ who indicted a higher sensitivity of >90% and specificity of 70.8% in the assessment of focal breast lesions. -Although some of the above AI-based applications are approved in some countries, until now there are yet no guides to endorse the AI-based applications alongside US in daily clinical practice.^68^

## AI-based application and magnetic resonance imaging (MRI)

Breast MRI shows high sensitivity in breast cancer detection.^68^ Dynamic contrast material enhanced (DCE) MRI of the breast screening showed the highest sensitivity compared to the other imaging modalities (such as mammogram and US).^69^ However, false-negative results can be obtained with a reported sensitivity of 90.9% for invasive cancer and 73% for ductal carcinoma.^70^ Several studies reviewed breast MRI examinations retrospectively and found that some breast lesions had been either missed, misdiagnosed, or mismanaged.^71,72^ This may be attributed to the various factors such as dense breast tissue, visual examination pattern, incorrect assessment, image quality, distraction, fatigue, workload, poor enhancement, or misdiagnose enhancement.^73-75^ Automated detection of breast carcinomas in MRI images via AI-based applications (such as CNN) has been indicated for systematic diagnostic interpretation and identification of tumors on images stored on an archiving system.^76,77^

Literature reported the utilization of DL for MRI breast screening^75,78-81^ and most popular role of the proposed AI-based applications have included tasks, such as detection, segmentation, and classification of lesions in MRI images.^75,78,82-84^ A decent performance was reported by Truhn et al,^85^ who used CNN to categorize segmented lesions as benign or malignant, reached AUC of 0.88, which was superior to radiomics analysis (AUC=0.81); however, this was inferior to the breast radiologist’s interpretation (AUC = 0.98).^85^ Similarly reported by Herent et al,^80^ who used the DL model to identify and categorize breast lesions as benign or malignant, achieving an AUC of 0.816. Ayatollahi et al^86^ promoted a DL model for the detection of breast lesions in ultrafast DCE-MRI sequences. This proposed model achieved a high detection rate of 0.90 (0.876-0.934), sensitivity of 0.95 (0.934–0.980), and a detection rate of benign lesions of 0.81 (0.751-0.871).

An improvement in radiologists’ clinical performance with the assistance of an AI tool was also reported by Adachi et al.^81^ A study carried out by Eskreis-Winkler et al^87^ showed improvement in accuracy (92.8%), sensitivity (89.5%), and specificity (94.3%) of detection of breast cancer with the assistance of an AI tool. They also found that using DL tools could lead to a reduction in reading time (3 and 45 seconds per case).^87^ This was consistent with Jiang et al,^88^ who evaluated the clinical performance of radiologists in detecting breast cancer at DCE MRI, and found that AUC showed an improvement in accuracy from 0.71 to 0.76 when AI was used.^69^ This was reinforced recently by Wu et al,^89^ who found that the proposed CNN model based on DCE MRI achieved diagnostic accuracy of 87.7%, precision of 91.2%, sensitivity of 86.1%, and AUC of 91.2%. In addition, it was highlighted that DL can be a favorable tool to increase the proficiency and accessibility of breast MRI.^88^ Jing et al^88^ found that the proposed AI model achieved an AUC of 0.81 with a 15.7% reduction in workload and a 16.6% reduction in scanning time.

In addition, AI and radiomics approach have gained popularity in medical imaging to facilitate disease diagnosis (such as breast lesions).^89,90^ In an MRI-based radiomics and AI study, entropy of breast lesions found to be a worth parameters to differentiate between malignant and benign breast lesions.^90,91^ Fusco et al^92^ reported consistent findings. Another study carried out by Crivelli et al^93^ revealed that radiomics values were lower than those suspected by expert breast radiologists. Thus, it is essential to notice that the promising evidence of radiomics still requires further evaluation and many issues need to be solved prior to it being ready to be implemented in clinical practice.

The above studies show that AI-based applications are a promising tool for breast image screening and cancer detection. However, several challenges and limitations need to be considered. Picture Archiving and Communication Systems (PACS) and the Digital Imaging and Communications in Medicine (DICOM) have ensured that datasets required to train and test AI-based applications are prepared for easy access and recovery. Yet, organizing the datasets (labeling, annotation, segmentation) represents a major issue in developing AI-based applications as trained professionals (clinical scientists and informaticians) are required and this is a time-consuming and high-cost process.^94-96^ Comparing the clinical value of different AI-based applications is challenging due to the variation in the datasets, the approach of testing, and validation of the tool performance.^39,97,98^ This is combined with the lack of high-quality, categorized, labeled datasets, which are representative as well as including a good distribution of abnormalities, demographics, and breast density.^18^ Inappropriate datasets and poor image quality may limit the conspicuity of the breast lesion’s characters or offer inadequate inputs for the AI system.^99,100^

Although the reviewed studies in this article reported encouraging findings of the proposed AI model’s accuracy, most of these studies were retrospective, based on relatively small datasets, came from single institutions, and identified several methodological restrictions that negatively impact on the applicability and strength of the AI-based applications in the breast screening setting. Using small datasets from the same source may raise concerns regarding the depth, quality, and representativeness of the images that are used to teach the AI-based applications and increase the possibility of bias and overfitting. In addition, datasets enriched with malignant lesions or suspicious abnormalities were also used to train the AI tool. This approach may assist the feasibility of designing and developing an AI tool; conversely, the applicability and accuracy of the proposed AI tool performance will still be unclear or overestimated as the datasets may not reflect the real-world screening of detectable breast cancer. Several factors may affect the performance of the AI-based applications, in particular patient populations such as heterogeneity of the breast cancer risk factors, and imaging characters of the populations. Therefore, a larger validation dataset from diverse screening environments and populations is required to ensure that AI tools are ready for use in real clinical practice.

In conclusion, this review of the available research on AI-based applications and breast cancer screening provides insight into the value of AI tools combined with diverse imaging modalities in breast lesion detection and diagnosis. The shift from the conventional CAD system to the advanced AI tools such as DL-CAD has the potential to reduce false-positive findings, increase diagnostic accuracy, improve radiologist performance, and assist with decision-making. However, the current evidence regarding the use of AI-based applications in the detection of the breast cancer is not yet fully optimized due to a lack of standardized methodology and prospective studies, the possibility of bias, and a lack of depth and quality. Future randomized controlled trials and cohort studies in large-scale sample with high-quality evidence are required to consider the future use of AI-based applications in breast cancer screening.
